# Supplementary material for: Does Carrying a Rider Change Motor and Sensory Laterality in Horses?
Source: Animals (Basel). 2022 Apr 12;12(8):992. doi: 10.3390/ani12080992 (PMC9027692; doi:10.3390/ani12080992)
Supplement: Supplementary file 1 [file animals-12-00992-s001.zip › animals-1632543-supplementary.pdf]

File S1. List of Novel Objects.

Sensory Laterality Object Test

Novel Objects as chosen by the horses' owners:

- Pink gymnastic ball
- Red umbrella
- Wooden sled
- Pink bag
- Green stool
- Blue and yellow balloons
- Green bag
- Orange umbrella
- Green children wheelbarrow
- White plastic chair
- Car tire
- Multi-coloured plastic bag
- Brown basket
- Black safety vest
- Multi-coloured pillow
- Blue umbrella
- Red Bobbycar
- Silver windscreen cover
- Grey rubbish bag
- Green scooter
- Orange children's play tunnel
- Inline skates
- Multi-coloured blanket
- Pool noodle
- Green barrel
- Multi-coloured umbrella
- Green chair
- Green ball
- Red plastic bag
- Blue barrel
- Hazzard tape
- Empty plastic bottles
- Tin foil
- Wooden basket
- Plastic sandbox toys
- Gift wrapping
- Doll
- Watering can

File S2. Statistical Data.

Difference in strength of laterality (absolute LI value) on population level without a rider in comparison to with a passive rider?

Motor Laterality

```
Rcmdr>  GLM.23 <- glm(ML_ABS ~ W.WO.Rider + age + breed + training +
Rcmdr+    side.of...leading + sex, family=gaussian(identity), data=Dataset)

Rcmdr>  summary(GLM.23)

Call:
glm(formula = ML_ABS ~ W.WO.Rider + age + breed + training +
    side.of...leading + sex, family = gaussian(identity), data = Dataset)

Deviance Residuals:
     Min        1Q    Median        3Q       Max
-0.44429  -0.15008  -0.00636   0.17003   0.66991

Coefficients:
                            Estimate Std. Error t value Pr(>|t|)
(Intercept)                0.2105529  0.1723748   1.221   0.2296
W.WO.Rider[T.With Rider]   0.1913043  0.0732465   2.612   0.0129 *
age                        0.0037207  0.0062871   0.592   0.5576
breed[T.draught horse]    -0.1594452  0.1493921  -1.067   0.2928
breed[T.pony]             -0.0355526  0.1058317  -0.336   0.7388
breed[T.thoroughbred]      0.0939209  0.1177041   0.798   0.4300
training                   0.0008157  0.0235237   0.035   0.9725
side.of...leading[T.left] -0.0895081  0.1023143  -0.875   0.3873
sex[T.mare]                0.1766685  0.0964912   1.831   0.0752 .
---
Signif. codes:  0 '***' 0.001 '**' 0.01 '*' 0.05 '.' 0.1 ' ' 1

(Dispersion parameter for gaussian family taken to be 0.06169809)

    Null deviance: 2.9983  on 45  degrees of freedom
Residual deviance: 2.2828  on 37  degrees of freedom
AIC: 12.394

Number of Fisher Scoring iterations: 2
```

```
Rcmdr>  GLM.24 <- glm(ML_ABS ~ W.WO.Rider + age + breed +  side.of...leading + sex,
```

```
Rcmdr+    family=gaussian(identity), data=Dataset)

Rcmdr>  summary(GLM.24)

Call:
glm(formula = ML_ABS ~ W.WO.Rider + age + breed + side.of...leading +
    sex, family = gaussian(identity), data = Dataset)

Deviance Residuals:
     Min        1Q    Median        3Q       Max
-0.44613  -0.15075  -0.00628   0.17123   0.66982

Coefficients:
                           Estimate Std. Error t value Pr(>|t|)
(Intercept)                0.215154   0.108568   1.982   0.0548 .
W.WO.Rider[T.With Rider]   0.191304   0.072277   2.647   0.0118 *
age                        0.003735   0.006190   0.603   0.5498
breed[T.draught horse]    -0.157603   0.137777  -1.144   0.2598
breed[T.pony]             -0.033996   0.094566  -0.359   0.7212
breed[T.thoroughbred]      0.093543   0.115647   0.809   0.4236
side.of...leading[T.left] -0.091223   0.088387  -1.032   0.3086
sex[T.mare]                0.176490   0.095078   1.856   0.0712 .
---
Signif. codes:  0 '***' 0.001 '**' 0.01 '*' 0.05 '.' 0.1 ' ' 1

(Dispersion parameter for gaussian family taken to be 0.06007641)

    Null deviance: 2.9983  on 45  degrees of freedom
Residual deviance: 2.2829  on 38  degrees of freedom
AIC: 10.395

Number of Fisher Scoring iterations: 2
```

```
Rcmdr>  GLM.25 <- glm(ML_ABS ~ W.WO.Rider + age +  side.of...leading + sex,
Rcmdr+    family=gaussian(identity), data=Dataset)

Rcmdr>  summary(GLM.25)

Call:
glm(formula = ML_ABS ~ W.WO.Rider + age + side.of...leading +
    sex, family = gaussian(identity), data = Dataset)
```

```
Deviance Residuals:
     Min        1Q    Median        3Q       Max
-0.38513  -0.18603   0.00618   0.17848   0.65644

Coefficients:
                           Estimate Std. Error t value Pr(>|t|)
(Intercept)                0.194397   0.104565   1.859   0.0702 .
W.WO.Rider[T.With Rider]   0.191304   0.071572   2.673   0.0107 *
age                        0.003464   0.006071   0.571   0.5715
side.of...leading[T.left] -0.055994   0.083188  -0.673   0.5047
sex[T.mare]                0.127640   0.087036   1.467   0.1501
---
Signif. codes:  0 '***' 0.001 '**' 0.01 '*' 0.05 '.' 0.1 ' ' 1

(Dispersion parameter for gaussian family taken to be 0.05890908)

    Null deviance: 2.9983  on 45  degrees of freedom
Residual deviance: 2.4153  on 41  degrees of freedom
AIC: 6.9882

Number of Fisher Scoring iterations: 2
```

```
Rcmdr>  GLM.26 <- glm(ML_ABS ~ W.WO.Rider +  side.of...leading + sex,
Rcmdr+    family=gaussian(identity), data=Dataset)

Rcmdr>  summary(GLM.26)

Call:
glm(formula = ML_ABS ~ W.WO.Rider + side.of...leading + sex,
    family = gaussian(identity), data = Dataset)

Deviance Residuals:
     Min        1Q    Median        3Q       Max
-0.37701  -0.18353   0.01429   0.17371   0.62299

Coefficients:
                          Estimate Std. Error t value Pr(>|t|)
(Intercept)                0.23281    0.07935   2.934  0.00541 **
W.WO.Rider[T.With Rider]   0.19130    0.07099   2.695  0.01009 *
side.of...leading[T.left] -0.04710    0.08106  -0.581  0.56427
sex[T.mare]                0.12922    0.08629   1.497  0.14175
---
```

```
Signif. codes:  0 '***' 0.001 '**' 0.01 '*' 0.05 '.' 0.1 ' ' 1

(Dispersion parameter for gaussian family taken to be 0.05796298)

    Null deviance: 2.9983  on 45  degrees of freedom
Residual deviance: 2.4344  on 42  degrees of freedom
AIC: 5.3519

Number of Fisher Scoring iterations: 2
```

```
Rcmdr>  GLM.27 <- glm(ML_ABS ~ W.WO.Rider +  sex, family=gaussian(identity),
Rcmdr+    data=Dataset)

Rcmdr>  summary(GLM.27)

Call:
glm(formula = ML_ABS ~ W.WO.Rider + sex, family = gaussian(identity),
    data = Dataset)

Deviance Residuals:
     Min        1Q    Median        3Q       Max
-0.39010  -0.19010   0.00121   0.20121   0.60990

Coefficients:
                         Estimate Std. Error t value Pr(>|t|)
(Intercept)               0.19879    0.05316   3.740  0.00054 ***
W.WO.Rider[T.With Rider]  0.19130    0.07045   2.716  0.00949 **
sex[T.mare]               0.12556    0.08539   1.470  0.14876
---
Signif. codes:  0 '***' 0.001 '**' 0.01 '*' 0.05 '.' 0.1 ' ' 1

(Dispersion parameter for gaussian family taken to be 0.05707022)

    Null deviance: 2.9983  on 45  degrees of freedom
Residual deviance: 2.4540  on 43  degrees of freedom
AIC: 3.7203

Number of Fisher Scoring iterations: 2
```

Rcmdr> GLM.28 <- glm(ML_ABS ~ W.WO.Rider, family=gaussian(identity), data=Dataset)

```
Rcmdr>  summary(GLM.28)

Call:
glm(formula = ML_ABS ~ W.WO.Rider, family = gaussian(identity), 
    data = Dataset)

Deviance Residuals: 
     Min        1Q    Median        3Q       Max  
-0.41739  -0.21739  -0.02174   0.17391   0.58261  

Coefficients:
                         Estimate Std. Error t value  Pr(>|t|)    
(Intercept)               0.22609    0.05047    4.48 0.0000526 ***
W.WO.Rider[T.With Rider]  0.19130    0.07137    2.68    0.0103 *  
---
Signif. codes:  0 '***' 0.001 '**' 0.01 '*' 0.05 '.' 0.1 ' ' 1 

(Dispersion parameter for gaussian family taken to be 0.05857708)

    Null deviance: 2.9983  on 45  degrees of freedom
Residual deviance: 2.5774  on 44  degrees of freedom
AIC: 3.9766

Number of Fisher Scoring iterations: 2
```

```
Rcmdr>  anova(GLM.28, GLM.27, test="F")
Analysis of Deviance Table

Model 1: ML_ABS ~ W.WO.Rider
Model 2: ML_ABS ~ W.WO.Rider + sex
  Resid. Df Resid. Dev Df Deviance      F Pr(>F)
1        44     2.5774                          
2        43     2.4540  1  0.12337 2.1618 0.1488
```

Result: Significant difference in strength of motor laterality; horses showed stronger motor laterality with a passive rider than without.

Sensory Laterality Novel Object Test

```
Rcmdr>  GLM.30 <- glm(NO_ABS ~ W.WO.Rider + training + side.of...leading + sex +
Rcmdr+    age + breed, family=gaussian(identity), data=Dataset)

Rcmdr>  summary(GLM.30)

Call:
glm(formula = NO_ABS ~ W.WO.Rider + training + side.of...leading +
    sex + age + breed, family = gaussian(identity), data = Dataset)

Deviance Residuals:
     Min        1Q    Median        3Q       Max
-0.47205  -0.24158  -0.08649   0.30006   0.67263

Coefficients:
                            Estimate Std. Error t value Pr(>|t|)
(Intercept)                0.7234006  0.2377449   3.043   0.0043 **
W.WO.Rider[T.With Rider]   0.0289870  0.1010239   0.287   0.7758
training                   0.0003781  0.0324446   0.012   0.9908
side.of...leading[T.left] -0.1255022  0.1411152  -0.889   0.3796
sex[T.mare]               -0.0306433  0.1330837  -0.230   0.8192
age                       -0.0054962  0.0086713  -0.634   0.5301
breed[T.draught horse]     0.0838942  0.2060464   0.407   0.6862
breed[T.pony]             -0.1271128  0.1459665  -0.871   0.3895
breed[T.thoroughbred]     -0.2015395  0.1623413  -1.241   0.2223
---
Signif. codes:  0 '***' 0.001 '**' 0.01 '*' 0.05 '.' 0.1 ' ' 1

(Dispersion parameter for gaussian family taken to be 0.1173671)

    Null deviance: 5.0338  on 45  degrees of freedom
Residual deviance: 4.3426  on 37  degrees of freedom
AIC: 41.974

Number of Fisher Scoring iterations: 2
```

```
Rcmdr>  GLM.31 <- glm(NO_ABS ~ W.WO.Rider +  side.of...leading + sex + age + breed,
Rcmdr+    family=gaussian(identity), data=Dataset)
```

```
Rcmdr>  summary(GLM.31)

Call:
glm(formula = NO_ABS ~ W.WO.Rider + side.of...leading + sex + 
    age + breed, family = gaussian(identity), data = Dataset)

Deviance Residuals: 
     Min        1Q    Median        3Q       Max  
-0.47298  -0.24159  -0.08649   0.30016   0.67316  

Coefficients:
                           Estimate Std. Error t value  Pr(>|t|)    
(Intercept)                0.725533   0.149739   4.845 0.0000215 ***
W.WO.Rider[T.With Rider]   0.028987   0.099686   0.291     0.773    
side.of...leading[T.left] -0.126297   0.121905  -1.036     0.307    
sex[T.mare]               -0.030726   0.131133  -0.234     0.816    
age                       -0.005489   0.008537  -0.643     0.524    
breed[T.draught horse]     0.084748   0.190023   0.446     0.658    
breed[T.pony]             -0.126391   0.130427  -0.969     0.339    
breed[T.thoroughbred]     -0.201715   0.159502  -1.265     0.214    
---
Signif. codes:  0 '***' 0.001 '**' 0.01 '*' 0.05 '.' 0.1 ' ' 1

(Dispersion parameter for gaussian family taken to be 0.1142789)

    Null deviance: 5.0338  on 45  degrees of freedom
Residual deviance: 4.3426  on 38  degrees of freedom
AIC: 39.975

Number of Fisher Scoring iterations: 2
```

```
Rcmdr>  GLM.32 <- glm(NO_ABS ~ W.WO.Rider + side.of...leading + age + breed, 
Rcmdr+    family=gaussian(identity), data=Dataset)

Rcmdr>  summary(GLM.32)

Call:
glm(formula = NO_ABS ~ W.WO.Rider + side.of...leading + age + 
    breed, family = gaussian(identity), data = Dataset)

Deviance Residuals: 
     Min        1Q    Median        3Q       Max  
```

```
-0.46640  -0.23623  -0.08902   0.30184   0.65609

Coefficients:
                           Estimate Std. Error t value  Pr(>|t|)    
(Intercept)                0.724279   0.147819   4.900 0.0000172 ***
W.WO.Rider[T.With Rider]   0.028987   0.098471   0.294     0.770    
side.of...leading[T.left] -0.129990   0.119408  -1.089     0.283    
age                       -0.005560   0.008428  -0.660     0.513    
breed[T.draught horse]     0.073514   0.181634   0.405     0.688    
breed[T.pony]             -0.133608   0.125193  -1.067     0.292    
breed[T.thoroughbred]     -0.195751   0.155539  -1.259     0.216    
---
Signif. codes:  0 '***' 0.001 '**' 0.01 '*' 0.05 '.' 0.1 ' ' 1

(Dispersion parameter for gaussian family taken to be 0.1115096)

    Null deviance: 5.0338  on 45  degrees of freedom
Residual deviance: 4.3489  on 39  degrees of freedom
AIC: 38.041

Number of Fisher Scoring iterations: 2
```

```
Rcmdr>  GLM.33 <- glm(NO_ABS ~ W.WO.Rider + side.of...leading + age,
Rcmdr+    family=gaussian(identity), data=Dataset)

Rcmdr>  summary(GLM.33)

Call:
glm(formula = NO_ABS ~ W.WO.Rider + side.of...leading + age, 
    family = gaussian(identity), data = Dataset)

Deviance Residuals: 
    Min       1Q   Median       3Q      Max  
-0.4550  -0.1731  -0.1358   0.3232   0.5781  

Coefficients:
                           Estimate Std. Error t value  Pr(>|t|)    
(Intercept)                0.705003   0.142306   4.954 0.0000124 ***
W.WO.Rider[T.With Rider]   0.028987   0.098091   0.296     0.769    
side.of...leading[T.left] -0.183786   0.113764  -1.616     0.114    
age                       -0.004731   0.008317  -0.569     0.572    
---
```

```
Signif. codes:  0 '***' 0.001 '**' 0.01 '*' 0.05 '.' 0.1 ' ' 1

(Dispersion parameter for gaussian family taken to be 0.1106513)

    Null deviance: 5.0338  on 45  degrees of freedom
Residual deviance: 4.6474  on 42  degrees of freedom
AIC: 35.095

Number of Fisher Scoring iterations: 2
```

```
Rcmdr>  GLM.34 <- glm(NO_ABS ~ W.WO.Rider + side.of...leading,
Rcmdr+    family=gaussian(identity), data=Dataset)

Rcmdr>  summary(GLM.34)

Call:
glm(formula = NO_ABS ~ W.WO.Rider + side.of...leading, family = gaussian(identity),
    data = Dataset)

Deviance Residuals:
    Min       1Q   Median       3Q      Max
-0.4851  -0.1517  -0.1228   0.3188   0.5439

Coefficients:
                          Estimate Std. Error t value    Pr(>|t|)
(Intercept)                0.65217    0.10697   6.097 0.000000265 ***
W.WO.Rider[T.With Rider]   0.02899    0.09732   0.298      0.7672
side.of...leading[T.left] -0.19608    0.11081  -1.769      0.0839 .
---
Signif. codes:  0 '***' 0.001 '**' 0.01 '*' 0.05 '.' 0.1 ' ' 1

(Dispersion parameter for gaussian family taken to be 0.1089107)

    Null deviance: 5.0338  on 45  degrees of freedom
Residual deviance: 4.6832  on 43  degrees of freedom
AIC: 33.448

Number of Fisher Scoring iterations: 2
```

```
Rcmdr>  GLM.35 <- glm(NO_ABS ~ W.WO.Rider, family=gaussian(identity), data=Dataset)
```

```
Rcmdr>  summary(GLM.35)

Call:
glm(formula = NO_ABS ~ W.WO.Rider, family = gaussian(identity), 
    data = Dataset)

Deviance Residuals: 
    Min       1Q   Median       3Q      Max  
-0.5362  -0.2029  -0.1739   0.4638   0.4928  

Coefficients:
                         Estimate Std. Error t value      Pr(>|t|)    
(Intercept)               0.50725    0.07046   7.199 0.00000000584 ***
W.WO.Rider[T.With Rider]  0.02899    0.09965   0.291         0.772    
---
Signif. codes:  0 '***' 0.001 '**' 0.01 '*' 0.05 '.' 0.1 ' ' 1

(Dispersion parameter for gaussian family taken to be 0.1141855)

    Null deviance: 5.0338  on 45  degrees of freedom
Residual deviance: 5.0242  on 44  degrees of freedom
AIC: 34.681

Number of Fisher Scoring iterations: 2
```

```
Rcmdr>  anova(GLM.35, GLM.34, test="F")
Analysis of Deviance Table

Model 1: NO_ABS ~ W.WO.Rider
Model 2: NO_ABS ~ W.WO.Rider + side.of...leading
  Resid. Df Resid. Dev Df Deviance     F  Pr(>F)  
1        44     5.0242                            
2        43     4.6832  1    0.341 3.131 0.08391 .
---
Signif. codes:  0 '***' 0.001 '**' 0.01 '*' 0.05 '.' 0.1 ' ' 1
```

Result: No significant difference in the strength of sensory laterality in the novel object test.

Sensory Laterality Person Test

```
Rcmdr>  GLM.37 <- glm(Person_ABS ~ W.WO.Rider + training + side.of...leading + sex
Rcmdr+    + age + breed, family=gaussian(identity), data=Dataset)

Rcmdr>  summary(GLM.37)

Call:
glm(formula = Person_ABS ~ W.WO.Rider + training + side.of...leading +
    sex + age + breed, family = gaussian(identity), data = Dataset)

Deviance Residuals:
     Min        1Q    Median        3Q       Max
-0.68543  -0.22556   0.07285   0.22282   0.50839

Coefficients:
                           Estimate Std. Error t value Pr(>|t|)
(Intercept)                0.738614   0.212052   3.483  0.00129 **
W.WO.Rider[T.With Rider]  -0.017391   0.090106  -0.193  0.84801
training                   0.011574   0.028938   0.400  0.69149
side.of...leading[T.left] -0.062378   0.125865  -0.496  0.62311
sex[T.mare]               -0.198160   0.118702  -1.669  0.10348
age                        0.002435   0.007734   0.315  0.75467
breed[T.draught horse]     0.284026   0.183779   1.545  0.13074
breed[T.pony]             -0.279902   0.130192  -2.150  0.03817 *
breed[T.thoroughbred]     -0.041021   0.144797  -0.283  0.77853
---
Signif. codes:  0 '***' 0.001 '**' 0.01 '*' 0.05 '.' 0.1 ' ' 1

(Dispersion parameter for gaussian family taken to be 0.09337052)

    Null deviance: 5.0122  on 45  degrees of freedom
Residual deviance: 3.4547  on 37  degrees of freedom
AIC: 31.453

Number of Fisher Scoring iterations: 2
```

```
Rcmdr>  GLM.38 <- glm(Person_ABS ~ W.WO.Rider + training + side.of...leading + sex
Rcmdr+    + breed, family=gaussian(identity), data=Dataset)

Rcmdr>  summary(GLM.38)
```

```
Call:
glm(formula = Person_ABS ~ W.WO.Rider + training + side.of...leading +
    sex + breed, family = gaussian(identity), data = Dataset)

Deviance Residuals:
     Min        1Q    Median        3Q       Max
-0.71162  -0.22272   0.06393   0.21660   0.50872

Coefficients:
                          Estimate Std. Error t value Pr(>|t|)
(Intercept)                0.76169    0.19661   3.874 0.000409 ***
W.WO.Rider[T.With Rider]  -0.01739    0.08903  -0.195 0.846169
training                   0.01219    0.02853   0.427 0.671627
side.of...leading[T.left] -0.05447    0.12186  -0.447 0.657433
sex[T.mare]               -0.19671    0.11720  -1.678 0.101472
breed[T.draught horse]     0.28728    0.18130   1.585 0.121359
breed[T.pony]             -0.28386    0.12804  -2.217 0.032678 *
breed[T.thoroughbred]     -0.03915    0.14295  -0.274 0.785659
---
Signif. codes:  0 '***' 0.001 '**' 0.01 '*' 0.05 '.' 0.1 ' ' 1

(Dispersion parameter for gaussian family taken to be 0.09115693)

    Null deviance: 5.0122  on 45  degrees of freedom
Residual deviance: 3.4640  on 38  degrees of freedom
AIC: 29.576

Number of Fisher Scoring iterations: 2
```

```
Rcmdr>  GLM.39 <- glm(Person_ABS ~ W.WO.Rider +  side.of...leading + sex + breed,
Rcmdr+    family=gaussian(identity), data=Dataset)

Rcmdr>  summary(GLM.39)

Call:
glm(formula = Person_ABS ~ W.WO.Rider + side.of...leading + sex +
    breed, family = gaussian(identity), data = Dataset)

Deviance Residuals:
     Min        1Q    Median        3Q       Max
-0.69130  -0.21316   0.06404   0.24665   0.52489

Coefficients:
```

```
                          Estimate Std. Error t value Pr(>|t|)    
(Intercept)                0.83284    0.10338   8.056 7.97e-10 ***
W.WO.Rider[T.With Rider]  -0.01739    0.08809  -0.197   0.8445    
side.of...leading[T.left] -0.07949    0.10574  -0.752   0.4567    
sex[T.mare]               -0.19926    0.11581  -1.721   0.0933 .  
breed[T.draught horse]     0.31523    0.16730   1.884   0.0670 .  
breed[T.pony]             -0.26085    0.11493  -2.270   0.0288 *  
breed[T.thoroughbred]     -0.04466    0.14087  -0.317   0.7529    
---
Signif. codes:  0 '***' 0.001 '**' 0.01 '*' 0.05 '.' 0.1 ' ' 1

(Dispersion parameter for gaussian family taken to be 0.08924619)

    Null deviance: 5.0122  on 45  degrees of freedom
Residual deviance: 3.4806  on 39  degrees of freedom
AIC: 27.796

Number of Fisher Scoring iterations: 2
```

```
Rcmdr>  GLM.40 <- glm(Person_ABS ~ W.WO.Rider + sex + breed,
Rcmdr+    family=gaussian(identity), data=Dataset)

Rcmdr>  summary(GLM.40)

Call:
glm(formula = Person_ABS ~ W.WO.Rider + sex + breed, family = gaussian(identity),
    data = Dataset)

Deviance Residuals:
     Min        1Q    Median        3Q       Max
-0.69130  -0.17967   0.06735   0.23621   0.50416

Coefficients:
                         Estimate Std. Error t value Pr(>|t|)    
(Intercept)               0.77967    0.07498  10.398 6.19e-13 ***
W.WO.Rider[T.With Rider] -0.01739    0.08761  -0.198   0.8437    
sex[T.mare]              -0.21134    0.11407  -1.853   0.0713 .  
breed[T.draught horse]    0.33469    0.16439   2.036   0.0484 *  
breed[T.pony]            -0.26644    0.11407  -2.336   0.0246 *  
breed[T.thoroughbred]    -0.07098    0.13570  -0.523   0.6039    
---
Signif. codes:  0 '***' 0.001 '**' 0.01 '*' 0.05 '.' 0.1 ' ' 1
```

```
(Dispersion parameter for gaussian family taken to be 0.08827586)

    Null deviance: 5.0122  on 45  degrees of freedom
Residual deviance: 3.5310  on 40  degrees of freedom
AIC: 26.458

Number of Fisher Scoring iterations: 2
```

```
Rcmdr>  GLM.41 <- glm(Person_ABS ~ W.WO.Rider + breed, family=gaussian(identity),
Rcmdr+    data=Dataset)

Rcmdr>  summary(GLM.41)

Call:
glm(formula = Person_ABS ~ W.WO.Rider + breed, family = gaussian(identity),
    data = Dataset)

Deviance Residuals:
     Min        1Q    Median        3Q       Max
-0.69130  -0.19527   0.05284   0.25284   0.58870

Coefficients:
                         Estimate Std. Error t value Pr(>|t|)
(Intercept)               0.74716    0.07503   9.958 1.66e-12 ***
W.WO.Rider[T.With Rider] -0.01739    0.09018  -0.193  0.84802
breed[T.draught horse]    0.26154    0.16424   1.592  0.11897
breed[T.pony]            -0.31846    0.11379  -2.799  0.00778 **
breed[T.thoroughbred]    -0.03846    0.13850  -0.278  0.78264
---
Signif. codes:  0 '***' 0.001 '**' 0.01 '*' 0.05 '.' 0.1 ' ' 1

(Dispersion parameter for gaussian family taken to be 0.09351366)

    Null deviance: 5.0122  on 45  degrees of freedom
Residual deviance: 3.8341  on 41  degrees of freedom
AIC: 28.245

Number of Fisher Scoring iterations: 2
```

```
Rcmdr>  anova(GLM.41, GLM.40, test="F")
Analysis of Deviance Table
```

```
Model 1: Person_ABS ~ W.WO.Rider + breed
Model 2: Person_ABS ~ W.WO.Rider + sex + breed
  Resid. Df Resid. Dev Df Deviance      F  Pr(>F)
1        41     3.8341
2        40     3.5310  1  0.30303 3.4327 0.07131 .
---
Signif. codes:  0 '***' 0.001 '**' 0.01 '*' 0.05 '.' 0.1 ' ' 1

Rcmdr>  GLM.42 <- glm(Person_ABS ~ W.WO.Rider, family=gaussian(identity),
Rcmdr+    data=Dataset)

Rcmdr>  summary(GLM.42)

Call:
glm(formula = Person_ABS ~ W.WO.Rider, family = gaussian(identity),
    data = Dataset)

Deviance Residuals:
    Min       1Q   Median       3Q      Max
-0.6956  -0.2913   0.1130   0.3044   0.3217

Coefficients:
                         Estimate Std. Error t value Pr(>|t|)
(Intercept)               0.69565    0.07035   9.888 9.44e-13 ***
W.WO.Rider[T.With Rider] -0.01739    0.09949  -0.175    0.862
---
Signif. codes:  0 '***' 0.001 '**' 0.01 '*' 0.05 '.' 0.1 ' ' 1

(Dispersion parameter for gaussian family taken to be 0.113834)

    Null deviance: 5.0122  on 45  degrees of freedom
Residual deviance: 5.0087  on 44  degrees of freedom
AIC: 34.539

Number of Fisher Scoring iterations: 2
```

```
Rcmdr>  anova(GLM.42, GLM.41, test="F")
Analysis of Deviance Table

Model 1: Person_ABS ~ W.WO.Rider
Model 2: Person_ABS ~ W.WO.Rider + breed
  Resid. Df Resid. Dev Df Deviance     F  Pr(>F)
```

```
1        44     5.0087
2        41     3.8341  3   1.1746 4.187 0.01127 *
---
Signif. codes:  0 '***' 0.001 '**' 0.01 '*' 0.05 '.' 0.1 ' ' 1
```

```
Rcmdr>  Dataset <- readXL("C:/Users/isabe/Desktop/Mappe1.xlsx", rownames=FALSE,
Rcmdr+    header=TRUE, na="", sheet="Tabelle2", stringsAsFactors=TRUE)
RcmdrMsg: [13] HINWEIS: Die Datenmatrix 'Dataset' hat 23 Zeilen und 27 Spalten.

Rcmdr>  with(Dataset, tapply(ABS.Person.with.rider, breed, median, na.rm=TRUE))
draught horse          pony  thoroughbred     warmblood
          1.0           0.4           0.8           0.8

Rcmdr>  kruskal.test(ABS.Person.with.rider ~ breed, data=Dataset)

        Kruskal-Wallis rank sum test

data:  ABS.Person.with.rider by breed
Kruskal-Wallis chi-squared = 4.4311, df = 3, p-value = 0.2185


Rcmdr>  with(Dataset, tapply(ABS.Person.without.rider, breed, median, na.rm=TRUE))
draught horse          pony  thoroughbred     warmblood
          1.0           0.4           1.0           0.8

Rcmdr>  kruskal.test(ABS.Person.without.rider ~ breed, data=Dataset)

        Kruskal-Wallis rank sum test

data:  ABS.Person.without.rider by breed
Kruskal-Wallis chi-squared = 7.2812, df = 3, p-value = 0.06345
```

Result: No significant difference in the strength of sensory laterality in the person test.

The breed of the horses had a significant influence: Ponies were stronger lateralised than other breeds with and without rider.

Table S1. Data.

Leader number	Rider number	Horse number	Horse name	Presence Passive Rider
1		1	Roy	Without Rider
1	1	1	Roy	With Rider
1		2	Pesus	Without Rider
1	2	2	Pesus	With Rider
1		3	Donald	Without Rider
1	1	3	Donald	With Rider
1		4	Julia	Without Rider
1	3	4	Julia	With Rider
2		5	Silence	Without Rider
2	10	5	Silence	With Rider
2		6	Coco	Without Rider
2	10	6	Coco	With Rider
3		7	Fanta	Without Rider
3	11	7	Fanta	With Rider
3		8	Unca	Without Rider
3	11	8	Unca	With Rider
4		9	Heidi	Without Rider
4	4	9	Heidi	With Rider
4		10	Adonis	Without Rider
4	4	10	Adonis	With Rider
4		11	Jamie	Without Rider
4	4	11	Jamie	With Rider
5		12	Burly	Without Rider
5	5	12	Burly	With Rider
5		13	Lenny	Without Rider
5	5	13	Lenny	With Rider
5		14	Hobbit	Without Rider
5	5	14	Hobbit	With Rider
6		15	Clay	Without Rider
6	6	15	Clay	With Rider
7		16	Apolo	Without Rider
7	7	16	Apolo	With Rider
7		17	Capsius	Without Rider
7	7	17	Capsius	With Rider
8		18	Günther	Without Rider
8	9	18	Günther	With Rider
9		19	Dakkar	Without Rider
9	8	19	Dakkar	With Rider
9		20	Lucky	Without Rider
9	8	20	Lucky	With Rider
9		21	Don	Without Rider
9	8	21	Don	With Rider
7		22	Romero	Without Rider
7	7	22	Romero	With Rider

7		23	Alek	Without Rider
7	7	23	Alek	With Rider

Motor Laterality Index	Motor Laterality Index ABS
0	0,0
0,8	0,8
0,4	0,4
0,2	0,2
0,6	0,6
0,6	0,6
-0,2	0,2
-0,4	0,4
-0,2	0,2
-0,6	0,6
0	0,0
0,4	0,4
-0,6	0,6
-1	1,0
0	0,0
-0,4	0,4
0,4	0,4
0,4	0,4
0,4	0,4
-0,2	0,2
0	0,0
-0,2	0,2
-0,2	0,2
0,6	0,6
-0,2	0,2
-0,6	0,6
-0,2	0,2
0	0,0
-0,2	0,2
-1	1,0
0	0,0
0	0,0
-0,4	0,4
-0,2	0,2
0,4	0,4
0,6	0,6
0,4	0,4
0,4	0,4
0	0,0
0,4	0,4
0	0,0
0,2	0,2
0,2	0,2
-0,4	0,4

0,2	0,2
0	0,0

Sensory Laterality Novel Object Index	Sensory Laterality Novel Object Index ABS
0,0	0,0
0,7	0,7
0,0	0,0
0,3	0,3
0,3	0,3
-0,3	0,3
1,0	1,0
0,0	0,0
-0,3	0,3
0,3	0,3
0,3	0,3
-0,3	0,3
-0,3	0,3
0,3	0,3
1,0	1,0
1,0	1,0
-0,3	0,3
1,0	1,0
-1,0	1,0
-0,3	0,3
-0,3	0,3
1,0	1,0
1,0	1,0
0,3	0,3
0,3	0,3
-0,3	0,3
0,3	0,3
1,0	1,0
-0,3	0,3
-1,0	1,0
-0,3	0,3
0,3	0,3
-0,3	0,3
-1,0	1,0
-1,0	1,0
-0,3	0,3
1,0	1,0
1,0	1,0
0,3	0,3
0,3	0,3
-0,3	0,3
-0,3	0,3
-1,0	1,0
-0,3	0,3

-0,3	0,3
0,3	0,3

Sensory Laterality Person Test Index	Sensory Laterality Person Test Index ABS	Sex
0,4	0,4	gelding
0,2	0,2	gelding
0,2	0,2	gelding
-0,6	0,6	gelding
1,0	1,0	gelding
1,0	1,0	gelding
0,0	0,0	mare
0,0	0,0	mare
-0,4	0,4	gelding
0,0	0,0	gelding
0,6	0,6	mare
0,4	0,4	mare
-0,8	0,8	mare
-0,8	0,8	mare
-1,0	1,0	gelding
-0,8	0,8	gelding
1,0	1,0	mare
1,0	1,0	mare
-1,0	1,0	gelding
-0,8	0,8	gelding
-1,0	1,0	gelding
-1,0	1,0	gelding
-0,4	0,4	mare
-0,2	0,2	mare
-0,4	0,4	gelding
-0,8	0,8	gelding
-0,6	0,6	gelding
-0,2	0,2	gelding
0,8	0,8	gelding
0,4	0,4	gelding
1,0	1,0	gelding
1,0	1,0	gelding
1,0	1,0	gelding
1,0	1,0	gelding
0,2	0,2	gelding
0,6	0,6	gelding
0,8	0,8	gelding
1,0	1,0	gelding
0,8	0,8	gelding
1,0	1,0	gelding
0,6	0,6	gelding
1,0	1,0	gelding
-1,0	1,0	gelding
-1,0	1,0	gelding

1,0	1,0	gelding
0,8	0,8	gelding

Breed	Age (years)	Training (days per week)	Side of leading
pony	14	6,0	left
pony	14	6,0	left
warmbloo	23	1,5	left
warmbloo	23	1,5	left
thoroughb	29	4,0	left
thoroughb	29	4,0	left
pony	21	7,0	left
pony	21	7,0	left
thoroughb	4	5,0	left
thoroughb	4	5,0	left
pony	6	4,0	left
pony	6	4,0	left
warmbloo	14	6,0	both
warmbloo	14	6,0	both
warmbloo	6	6,0	both
warmbloo	6	6,0	both
draught ho	13	7,0	left
draught ho	13	7,0	left
warmbloo	20	7,0	left
warmbloo	20	7,0	left
draught ho	16	7,0	both
draught ho	16	7,0	both
warmbloo	14	2,0	left
warmbloo	14	2,0	left
warmbloo	11	4,0	left
warmbloo	11	4,0	left
warmbloo	16	3,5	left
warmbloo	16	3,5	left
warmbloo	4	3,5	left
warmbloo	4	3,5	left
warmbloo	9	6,0	left
warmbloo	9	6,0	left
warmbloo	11	2,5	left
warmbloo	11	2,5	left
pony	7	6,0	both
pony	7	6,0	both
warmbloo	12	7,0	both
warmbloo	12	7,0	both
pony	13	7,0	left
pony	13	7,0	left
warmbloo	12	7,0	both
warmbloo	12	7,0	both
warmbloo	16	1,0	left
warmbloo	16	1,0	left

thoroughb	10	1,0	left
thoroughb	10	1,0	left
